# Immuno-localization of definitive hematopoietic stem cells in the vascular niche of mouse fetal liver

**DOI:** 10.1016/j.xpro.2022.101580

**Published:** 2022-10-12

**Authors:** Atreyi Biswas, Shailendra Kumar Singh, Gayathri M. Kartha, Satish Khurana

**Affiliations:** 1 Stem Cells and Development Lab, School of Biology, Indian Institute of Science Education and Research Thiruvananthapuram, Maruthamala Campus, Vithura, Kerala 695551, India

**Keywords:** Developmental biology, Microscopy, Antibody, Stem Cells

## Abstract

Understanding the murine fetal liver (FL) hematopoietic microenvironment, which promotes HSC proliferation, warrants identifying innate relationships between stem cells and the niche. An inclusive study of these cell associations remains elusive. Here, we optimized a protocol to immunolabel HSCs alongside the FL vasculature, a promising niche component. We provide a comprehensive plan from tissue processing, immunohistochemistry, and confocal microscopy, to three-dimensional distance analyses between HSCs and vasculature. This technique can be adapted for achieving congruous outcomes for other cell types.

For complete details on the use and execution of this protocol, please refer to Biswas et al. (2020).

## Highlights

• Detailed immunolabeling approach to localize HSCs in E14.5 fetal liver
• Single photon imaging of fetal liver HSCs in vascular niche
• Three-dimensional cell association studies between HSCs and vascular niche
• Application of reporter line-independent characterization of fetal liver niche

## Abstract (editor highlights)

Publisher’s note: Undertaking any experimental protocol requires adherence to local institutional guidelines for laboratory safety and ethics.

## Graphical abstract

**Figure:**
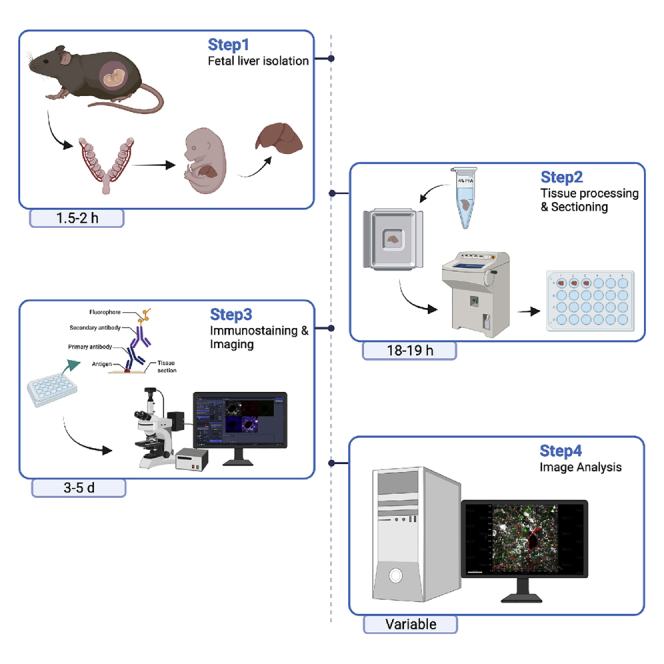

## Before you begin

### The experimental framework

Reliable identification and localization of HSCs is based on a number of important considerations, such as developmental stage, chosen markers, reagents and imaging tools used. Here, we describe a few factors that would be important for an optimal output and expanding the use of this method in a variety of target cell types in diverse tissues.

1. *A model for HSC proliferation*: Adult HSC division is tightly regulated to maintain a predominantly quiescent state, protecting the stem cell pool from stress factors like replication (Morrison and Scadden, 2014). Adult HSC divisions are sparse and temporally spaced out; lessons from developmental hematopoiesis, therefore, could be invaluable for understanding stem cell expansion. During murine hematopoietic development, blood formation starts in the yolk sac (YS) and placenta, followed by several transient hematopoietic sites (Medvinsky et al., 2011). Definitive hematopoiesis in embryo proper starts in aorta gonad mesonephros (AGM), followed by appearance in extra-embryonic vessels (EEVs), and fetal liver (FL), before finally colonizing the marrow of developing bones. Upon migration of HSCs and ingress into the FL, multiple fold expansion (Ema and Nakauchi, 2000) and concomitant maturation ensues. The prolific expansion events are supported well and do not lead to decline of HSC function (Biswas et al., 2020). Understanding the mechanisms underlying this controlled and effective stem cell proliferation is paramount in the field, for development and recapitulation in regenerative therapeutics. Although FL HSCs are intrinsically hardwired to constitute distinct characteristics compared to adult bone marrow HSCs (Copley and Eaves, 2013), the lesser unraveled FL HSC microenvironment harbors the potential to tolerate stem cell proliferation.

There are limited studies elucidating cell associations between HSCs and niche compartments within the FL. Khan et al. (2016), showed fractal-like increase patterns in the number of Nestin^+^NG2^+^ mural cells, FL HSCs and the surface area of portal vessels (Khan et al., 2016). Another study by Tamplin et al. (2015), demonstrated remodeling of vasculature, in murine FL and zebrafish caudal hepatic tissue, to accommodate HSCs (Tamplin et al., 2015). Both these studies underscored the relevance of vessels within the FL HSC microenvironment. A recent work by Lee Y et al., 2020, demonstrated the role of SCF from hepatic stellate cells, and emphasized the role of sinusoidal vessels in FL niche creation (Lee et al., 2020). Spatial transcriptomics has paved the way for improving our composite understanding of the FL HSC niche. Studies by Lu Y et al. and Gao S et al., 2021, highlight endothelial cells and macrophages, respectively, in the maintenance of HSCs within the FL (Lu et al., 2021; Gao et al., 2021). We recently reported the contribution of FL vasculature as HSC niche component, as endothelial cells were the main source of POSTN identified as a regulator of HSC proliferation (Biswas et al., 2020).

2. HSC reporter versus HSC immunohistochemical detection: Several reporter lines have been developed over the recent years that label HSCs, with varying degrees of purity, in the adult hematopoietic system. α-catulin^GFP/+^ (Acar et al., 2015) and Mds1^GFP/+^Flt3^Cre^ (Christodoulou et al., 2020) mice are some examples of reliable HSC reporters. Although the leaky expression of reporter lines is unavoidable, efforts have been made to improve the specificity of detection of at least a broad HSPC population. The Hlf reporter that largely selects a wider HSPC population in E14.5 FL is a noteworthy mention (Yokomizo et al., 2019), apart from the Hoxb4-YFP (Hills et al., 2011) and Ly-6A-GFP (de Bruijn et al., 2002) reporter mice that helps to identify all adult mouse HSPCs. Our technique would enable studies such as identification of cell microenvironments, in the absence of a suitably effective mouse reporter line.
3. Labelling murine HSCs and fetal liver vasculature: Major advances have been made in HSC identification through complex combinations of cell surface markers. Highly enriched HSC population (lin^-^c-Kit^+^Sca-1^+^CD150^+^CD48^-^CD244^-^) was identified using SLAM family receptors (Kiel et al., 2005). Limited by the number of fluorophores that can be detected without spectral overlap, we labeled HSCs using a cocktail of antibodies that we refer to as 3^-^ (lineage, CD41 and CD48) and Sca-1^+^. Our flow cytometry experiments showed that 3^-^Sca-1^+^ cells mark 75.02 ± 5.35% of the most primitive FL HSCs (data not shown). We label the total endothelial content, within the FL, using pan-endothelial marker CD31 (Albelda et al., 1991; Garcia-Porrero et al., 1998), while Lyve-1 is used to specifically mark sinusoidal endothelial cells (Nonaka et al., 2007). Every marker is individually tested for specific signals before being introduced into the antibody cocktail.
4. Choice of imaging platform: To acquire cellular information available across the FL tissue volume, it is necessary to sample through focal planes that are 1 μm apart, in the z-axis. We use confocal microscope Zeiss Axio Imager.Z2 for volumetric imaging of immunolabeled FL tissue. Other commercially available confocal microscopes can also be employed for this purpose. Imaging is performed using Plan-Apochromat 63× oil immersion objective. Photomultiplier tube is used for detection. Four-channel imaging is carried out and emission slider values are fine tuned to minimize the extent of bleed-through during image acquisition. Fluorophores are chosen with distinct emission ranges. We used AF405/ Hoechst 33342, AF488, AF594 and AF647 for labeling various subsets of cells. Care should be taken to choose fluorophores that can withstand 4–5 h of extensive laser exposure and are relatively more photo-stable.
5. Image Analysis: Physical closeness to the individual niche components has been used as an important criterion to predict the components of physical microenvironment of HSCs in FL. Eventually, the studies would lead to understand the molecular regulatory network underlying HSC function. Within the tissue, cells that are nearer to each other, can govern changes more effectively. In order to comprehend how associated a pair of cells are, we obtained the shortest distances or the three dimensional Euclidean distances between a pair of cells. We use commercially available software package Imaris (Oxford Instruments) for analyzing the data generated from confocal microscopy. We also use an open-source MATLAB code for generating random dots.

### Institutional permissions

All the methods used in this manuscript are in compliance with the guidelines laid down by the Institutional Animal Ethics Committee (IAEC) of IISER Thiruvananthapuram. The animals are bred and maintained as per the guidelines approved by the Committee for the Purpose of Control and Supervision of Experiments on Animals (CPCSEA), Ministry of Environment and Forests, Government of India.

### Timed mating of C57BL/6J mice

**Timing: 15 days**

C57BL/6J mice are housed in a specific pathogen free (SPF) animal facility with 12 h–12 h light-dark cycle, and provided with autoclaved water and food *ad libitum*. Mice are bred in a manner that would facilitate accurate tracing of developmental stages of the growing embryo. In this study, murine fetuses were staged at embryonic day (E) 14.5. The proliferation of FL HSCs peak at E14.5 and a snapshot of the microenvironment, at this stage, would be vital.

6. Keep the stud male singly in isolator cages for at least 3 days before mating.
7. Weigh 8–12 weeks old female mice and introduce them to male cages in the evening, for mating.

***Note:*** A male:female mating ratio of 1:1 is followed. The initial weight of the female mice is noted down for later reference.

8. Separate the female mice from the male mouse cages the following morning. Note this as E0.5. This assumption is made as the exact time of breeding cannot be ascertained.
9. Check the female mice for vaginal plug. House the females with plugs, separately. Mark the days following E0.5, by an increment of 1 day. Observe the weights of female mice from E6.5 onward.
10. Mice exhibiting a steady rise in weight are considered to be pregnant.

***Note:*** Pregnant dams might display irregularities in the increase of weight. This could be due to developmental termination of some embryos.

### Preparation of buffers

**Timing: 3–4 h**

**Table:** Phosphate buffer saline (1× PBS)

Reagent	Final concentration (mM)	Amount
KCl	2.68 mM	0.1 g
KH_2_PO_4_	1.76 mM	0.12 g
NaCl	136.89 mM	4 g
Na_2_HPO_4_	8.066 mM	0.5725 g
Double distilled (dd) H_2_O	N/A	Add to 500 mL
**Total**	**N/A**	**500 mL**

***Note:*** Use HCl or NaOH to adjust the pH of 1× PBS to 7.4. 1× PBS can be stored at 24°C–26°C for up to a period of 2 months.

**Table:** Phosphate buffer saline-Tween 20 (1× PBST)

Reagent	Final concentration	Amount
Tween 20	0.1%	50 μL
1× PBS	N/A	Add to 50 mL
**Total**	**N/A**	**50 mL**

***Note:*** 1× PBST can be stored in 4°C for 2–3 weeks.

**Table:** 4% paraformaldehyde (PFA)

Reagent	Final concentration	Amount
PFA	4%	4 g
1× PBS	N/A	Add to 100 mL
**Total**	**N/A**	**100 mL**

***Note:*** Heat 80 mL 1× PBS to 60°C in a glass beaker. Add the measured out PFA into the heated 1× PBS. Stir to obtain a milky white solution. Add NaOH beads, while mixing the solution continuously, to dissolve the PFA in 1× PBS. Adjust the pH of the solution to 7.4, using concentrated HCl. Make up the volume to 100 mL with 1× PBS. Aliquot into 15 mL sterile polypropylene centrifuge tubes as 10 mL units and store at −20°C, to avoid multiple freeze thaw cycles. 4% PFA can be stored in −20°C for 2–3 weeks.

**CRITICAL:** PFA is volatile and a potential carcinogen. PFA fumes can cause irritation if the eyes, skin or nasal epithelium are exposed. PFA should be handled inside a fume hood. Protective gears like gloves, masks and goggles should be used while preparing PFA.

***Note:*** 4% PFA stock is stored at −20°C. PFA needs to be taken out for thawing just before use.

**Table:** 30% sucrose

Reagent	Final concentration	Amount
Sucrose	30%	30 g
1× PBS	N/A	Add to 100 mL
**Total**	**N/A**	**100 mL**

***Note:*** 30% sucrose can be stored at 24°C–26°C for up to a period of 2 months.

**Table:** 0.01% Avidin

Reagent	Final concentration	Amount
Avidin	0.01%	25 mg
1× PBS	N/A	Reconstitute in 250 mL
**Total**	**N/A**	**250 mL**

***Note:*** Aliquot 0.01% avidin working concentration into 1.5 mL microcentrifuge tubes as 1 mL units and store at −20°C, to avoid multiple freeze thaw cycles. Working concentration of avidin is stable at −20°C and can be stored for long term.

**Table:** 0.05% Biotin

Reagent	Final concentration	Amount
Biotin	0.05%	100 mg
1× PBS	N/A	Reconstitute in 200 mL
**Total**	**N/A**	**200 mL**

***Note:*** Aliquot 0.05% biotin working concentration into 1.5 mL microcentrifuge tubes as 1 mL units and store at −20°C, to avoid multiple freeze thaw cycles. Working concentration of biotin is stable at −20°C and can be stored for long term.

**Table:** Hoechst (10 μg/mL)

Reagent	Final concentration	Amount
Hoechst (10 mg/mL;1000×)	1×; 10 μg/mL	1 μL
1× PBS	N/A	999 μL
**Total**	**N/A**	**1 mL**

***Note:*** Reconstitute 100 mg Hoechst 33342 in 10 mL 1× PBS to obtain a 10 mg/mL stock. Aliquot the stock into 0.5 mL amber microcentrifuge tubes as 100 μL units and store at −20°C, to avoid multiple freeze thaw cycles. 10 mg/mL stock concentration as well as 10 μg/mL working concentration of Hoechst is stable at −20°C and can be stored for long term.

## Key resources table

**Table:** 

REAGENT or RESOURCE	SOURCE	IDENTIFIER
**Chemicals, Peptides, and Recombinant Proteins**		

Sodium chloride (NaCl)	Sigma-Aldrich	Cat#: S7653; CAS: 7647-14-5
Potassium chloride (KCl)	Sigma-Aldrich	Cat#: P9333; CAS: 7447-40-7
Potassium phosphate monobasic (KH_2_PO_4_)	Sigma-Aldrich	Cat#: P5655; CAS: 7778-77-0
Sodium phosphate dibasic (Na_2_HPO_4_)	Sigma-Aldrich	Cat#: S7907; CAS: 7558-79-4
Sodium hydroxide (NaOH)	Sigma-Aldrich	Cat#: S8045; CAS: 1310-73-2
Hydrochloric acid (HCl)	Sigma-Aldrich	Cat#: 320331; CAS: 7647-01-0
Paraformaldehyde (PFA)	Sigma-Aldrich	Cat#: P6148; CAS: 30525-89-4
Biotin	Sigma-Aldrich	Cat#: B4501; CAS: 58-85-5
Avidin	Sigma-Aldrich	Cat#: A9275; CAS: 1405-69-2
Sucrose	Merck Millipore EMPERTA	Cat#: 107687; CAS: 57-50-1
2-Methyl butane	Sigma-Aldrich	Cat#: M32631; CAS: 78-78-4
Tween-20	Sigma-Aldrich	Cat#: P9416; CAS: 9005-64-5
Hoechst 33342	Sigma-Aldrich	Cat#: B2261; CAS: 875756-97-1
Prolong Gold anti-fade mountant	Invitrogen	P36934
PolyFreeze^TM^	Sigma-Aldrich	P0091
Ethanol	N/A	N/A
Methanol (MeOH)	Sigma-Aldrich	Cat#: 494437-2L; CAS: 67-56-1
MilliQ	Millipore	ZRXQ015T0

**Experimental models: Organisms/strains**		

Mice: C57BL/6J, 2–3 months old males and females	Animal Care and Resource Centre, NCBS (Bangalore, India)	n/a

**Antibodies**		

**Primary antibodies**		
CD31	R&D Systems	Cat#: AF3628; RRID: AB_2161028
Sca-1 FITC	BD Pharmingen	Cat#: 557405; RRID: AB_396688
Ter119 AF647	BioLegend	Cat#: 116218; RRID: AB_528961
B220 APC	BioLegend	Cat#: 103212; RRID: AB_312997
CD3e AF647	BioLegend	Cat#: 100209; RRID: AB_389323
Gr-1 APC	BioLegend	Cat#: 108412; RRID: AB_313377
F4/80 biotin	BioLegend	Cat#: 123106; RRID: AB_893501
CD41 biotin	eBioscience	Cat#: 13-0411-82; RRID: AB_763484
CD48 APC	BD Pharmingen	Cat#: 562746; RRID: AB_2737765
Lyve-1 AF488	Invitrogen	Cat#: 53-0443-82; RRID: AB_1633415
Sca-1 biotin	R&D Systems	Cat#: BAM1226; RRID: AB_2070040
Ter119 PE	BioLegend	Cat#: 116207; RRID: AB_313708
B220 PE	BioLegend	Cat#: 103207; RRID: AB_312992
CD3e PE	BioLegend	Cat#: 100308; RRID: AB_312673
Gr-1 PE	BioLegend	Cat#: 108407; RRID: AB_313372
F4/80 APC	BioLegend	Cat#: 123116; RRID: AB_893481
CD41 PE	BD Pharmingen	Cat#: 558040; RRID: AB_397004
CD48 PE	BioLegend	Cat#: 103405; RRID: AB_313020
**Secondary antibodies**		
Alexa Fluor® 647 Streptavidin	Jackson ImmunoResearch	Cat#: 016-600-084; RRID: AB_2341101
Alexa Fluor® 647 AffiniPure Goat Anti-Armenian Hamster IgG (H+L)	Jackson ImmunoResearch	Cat#: 127-605-099; RRID: AB_2339000
Alexa Fluor® 594 AffiniPure Fab Fragment Donkey Anti-Goat IgG (H+L)	Jackson ImmunoResearch	Cat#: 705-587-003; RRID: AB_2340435
Alexa Fluor® 647 AffiniPure Fab Fragment Donkey Anti-Goat IgG (H+L)	Jackson ImmunoResearch	Cat#: 705-607-003; RRID: AB_2340439

**Other**		

50 mL sterile polypropylene centrifuge tubes	Thermo Scientific	339652
15 mL sterile polypropylene centrifuge tubes	Thermo Scientific	339650
Cell culture dish (100 mm)	Eppendorf	0030702115
Cell culture dish (60 mm)	Eppendorf	0030701119
Microcentrifuge tubes (2 mL)	Tarsons	500020
Microcentrifuge tubes (1.5 mL)	Tarsons	500010
Microcentrifuge tubes amber (0.5 mL)	Tarsons	500012
Glass beaker (250 mL)	Riviera	7010036
Cell culture plate, 24 well	Eppendorf	0030722116
Frosted microscopy slides	VWR International	474702
Coverslip no.1 square (18 × 18 mm)	VWR International	16004-308
PAP pen (5 mm tip width)	Sigma-Aldrich	Z377821
Cryotome blade	Thermo Scientific	3053835
Dumont #5/45 Bent-tip Forceps	FST	11251-35
Dumont #5 Straight Forceps	FST	11252-20
Graefe Forceps	FST	11050-10
Extra Fine Graefe Forceps	FST	11150-10
Fine Scissors-Sharp	FST	14060-11
Disposable base molds (15 × 15 × 5 mm)	Fisherbrand	22-363-553
Metal Base Mold (24 × 24 × 10 mm)	Leica	3803082
Revolver adjustable tube rotator	Labnet	H5600
2D rocker	IKA	0004003000
Mortar and pestle	N/A	N/A
Fine brush (size 2)	Isabey	6217
Specimen chuck 40 mm	Thermo Scientific	715720
Aluminum foil	N/A	N/A
Cryotome	Thermo Scientific	HM525 NX
Stereo zoom microscope	ZEISS	Stemi 508
Plan-Apochromat 63×/1.40 Oil DIC M27	ZEISS	420782-9900
Plan-Apochromat 10×/0.45 ∞/0.17	ZEISS	420640-9900
Fluorescent lamp	ZEISS	HXP 120V
Immersion oil	ZEISS	Immersol^TM^ 518F
Axio Imager.Z2	ZEISS	LSM880

**Software and algorithms**		

Zen Blue 2.3	www.zeiss.com	N/A
Imaris	imaris.oxinst.com	N/A
Creating spots from .csv (Matlab Xtension)	github.com/BIDCatUCSF/Create-Spots-From-Text	N/A

## Step-by-step method details

### E14.5 fetal liver isolation

**Timing: 1.5–2 h**

This section contains a detailed description of the procedures for E14.5-pregnant mouse sacrifice, uterine horn isolation and harvesting the FL tissue.

1. Sacrifice a pregnant mouse 14.5 days post coitus, by cervical dislocation.

***Note:*** An increase in weight, from the initial weight, by 5 g or more is a reliable indicator of pregnancy.

2. Wipe the abdomen of the mouse with 70% (v/v) ethanol. Lift up the skin with a pair of Graefe forceps and make an incision, along the midline, with fine scissors. Continue cutting through the skin, on either sides of the incision, to procure a C-shaped opening that is perpendicular to the midline of the mouse (Methods Video S1).
3. Holding with the Graefe forceps, make additional cuts in the peritoneum. This would expose the abdominal cavity and the uterine horns can be visualized (Figure 1B).

**Figure 1:**
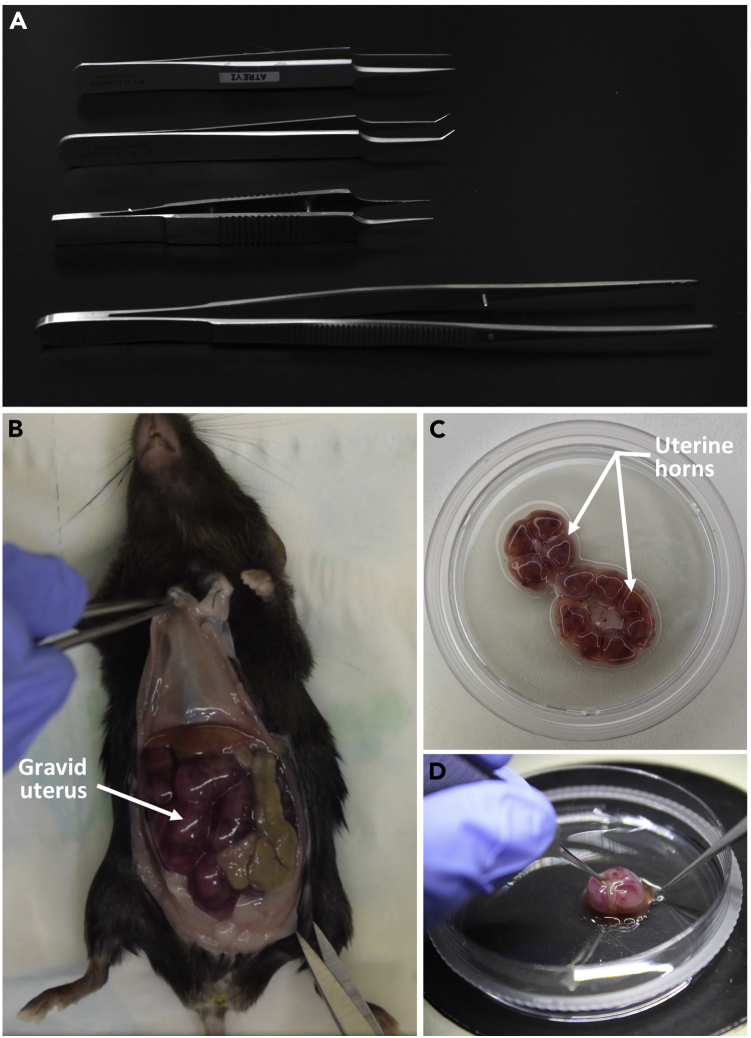
Harvesting E14.5 fetuses (A) Forceps used for mouse sacrifice and fetus dissection; from top to bottom: Extra Fine Graefe forceps, Dumont #5/45 Bent-tip forceps, Dumont #5 Straight forceps, Graefe forceps. (B) Exposed gravid uterus within the abdominal cavity of dissected E14.5 pregnant dam. (C) E14.5 uterine horns. (D) Individual E14.5 conceptus isolated from the uterine horn.
4. Hold the uterus with Graefe forceps and make cuts on the distal ends and the center to excise it out completely.
5. Transfer the uterine horns to a 50 mL sterile polypropylene centrifuge tube containing ice-cold 1× PBS, to remove excess blood (Methods Video S1).

**CRITICAL:** Maintain the tissues at 4°C.

6. Place the uterine horns in a 100 mm cell culture dish containing ice-cold 1× PBS. Using extra-fine Graefe forceps and Dumont #5 straight forceps, and working under a stereo zoom microscope, gently remove the endometrial tissue around each conceptus, taking care not to puncture the placenta (Figures 1A, 1C, and 1D).
7. Use the Dumont #5/45 bent-tip forceps to transfer the conceptus to a fresh 60 mm cell culture dish with ice-cold 1× PBS (Figure 2A).

**Figure 2:**
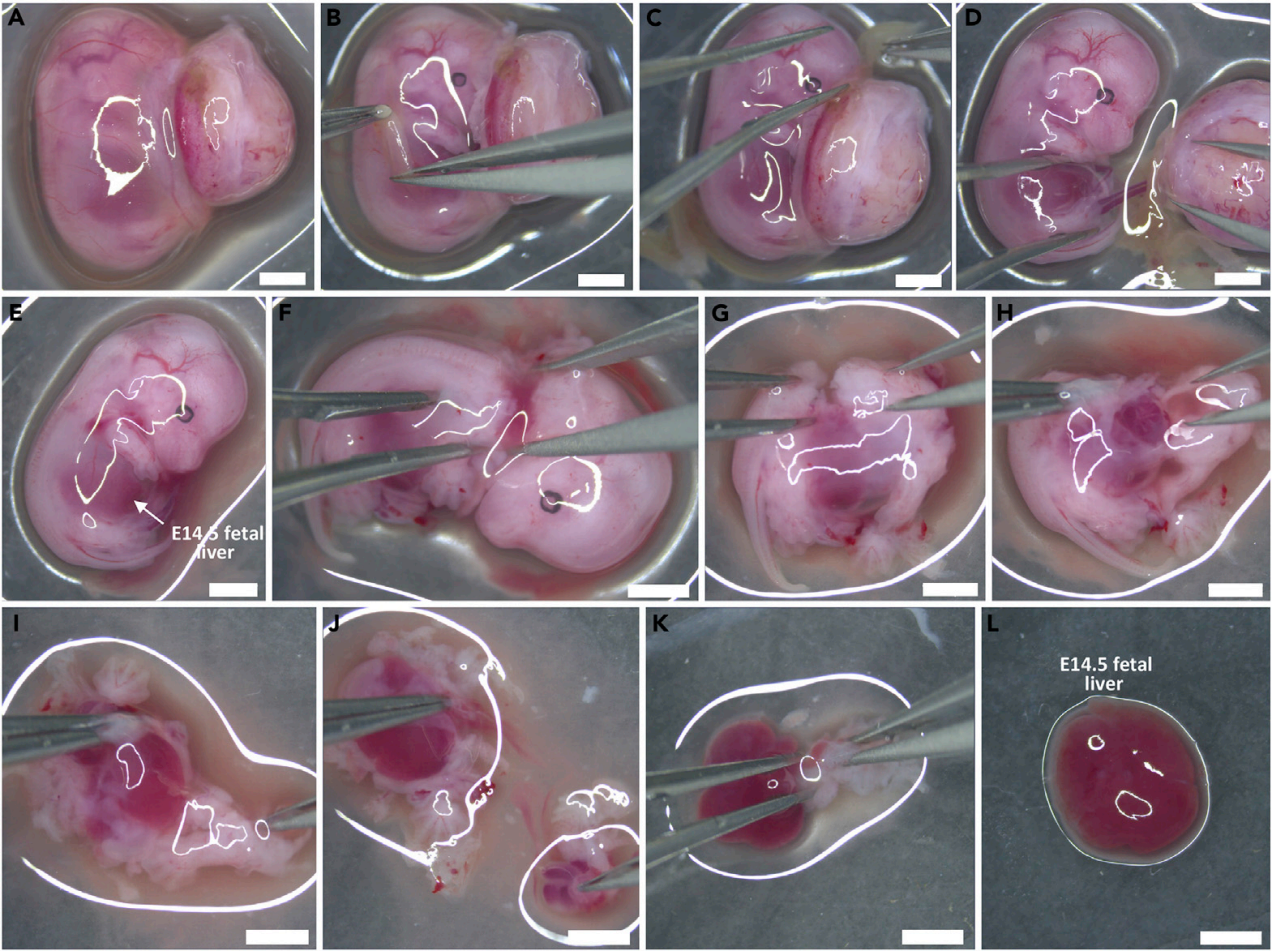
Steps to isolate E14.5 fetal liver (A) An E14.5 conceptus. (B) Incising YS around the fetus. (C) Complete removal of YS. (D) Nicking EEVs connecting the fetus and placenta. (E) Isolated E14.5 fetus. (F) Decapitation of the fetus. (G) Incising the dorsal tissue of the fetus. (H–K) Removal of tissues; dorsal and thoracic (H), dorsal and caudal (I), visceral organs (J), abdominal (K). (L) Isolated E14.5 FL. Scale bar: 2000 μm.
8. Use the Dumont #5 straight forceps to gently tease away the YS from the fetus, being careful not to nick any major blood vessel present in it (Figures 2B and 2C; Methods Video S2).

Methods Video S1. Harvesting uterine horns from pregnant dam, related to steps 2, 3, 4 and 5

9. Nip the EEVs connecting the fetus to the placenta (Figure 2D, Methods Video S2).

***Note:*** At E14.5 stage, the FL is a very prominent structure. Clearly visible under the epidermis, it is maroon coloured and present underneath the developing fetal diaphragm (Figure 2E).

10. Transfer the fetus to a new 60 mm cell culture dish with minimal level of ice cold 1× PBS, enough to keep the fetus wet. Optimal level of 1× PBS will make sure that the fetus remains securely in placed during dissection.
11. Use the extra fine Graefe forceps and Dumont #5 straight forceps to tease away fetal tissues from the rostral and caudal ends and expose the FL from within the abdominal cavity (Figures 2F–2L, Methods Video S2).

**CRITICAL:** Take utmost care not to damage the FL tissue while teasing away the extra abdominal tissues surrounding it. To this end, it is advisable to minimize or completely avoid direct handling of FL tissue with forceps.

***Note:*** After euthanizing the mice and dissection of tissues, all steps preceding fixation of tissue should be carried out quickly to minimize lysozyme mediated tissue degradation. Harvested tissues should be maintained on ice to reduce lysozyme activity.

Methods Video S2. Dissecting out murine E14.5 FL, related to steps 6, 7, 8, 9, 10 and 11

### Fixation, cryo-protection and cryo-block preparation

**Timing: ∼18 h**

This section contains detailed procedures for E14.5 FL fixation, cryo-protection and cryo-block preparation.

12. Use the Dumont #5/45 bent-tip forceps to transfer the FL tissues isolated into a 2 mL microcentrifuge tube containing 1.5 mL ice-cold 4% PFA.

**CRITICAL:** Take measures to transfer the tissue without causing damage.

***Note:*** A tissue:fixative volume ratio of approximately 1:20 is maintained to enable efficient fixation of each FL tissue. In order to maintain this ratio, one FL is added per 2 mL microcentrifuge tube, containing 1.5 mL 4% PFA.

13. Put the 2 mL microcentrifuge tubes, containing FLs, horizontally on ice and place it on a 2D rocker at 30 rpm, for 1 h of fixation.

***Note:*** The speed of the 2D rocker should enable sufficient mixing of PFA solution within the tube to prevent it from getting depleted at a particular end.

14. After 1 h, remove the PFA solution completely and add 1.5 mL 1× PBS and give a quick rinse. Discard this 1.5 mL 1× PBS immediately and add 1.5 mL 1× PBS again and wash the tissues for 30 min, placing the 2 mL tubes horizontally on ice, on the 2D rocker.

**CRITICAL:** It is important to remove the fixative after 1 h to prevent over-fixation of the FL tissue. The first quick wash with 1× PBS ensures that maximum amount of fixative is removed.

15. Give two more washes of 30 min each with 1× PBS, discarding 1× PBS from the previous wash and adding fresh 1× PBS each time. Add fresh 1× PBS to the tubes, at the end of three 30 min washes.

**CRITICAL:** Make sure that the pipette tip does not damage the tissue during change of fixative and wash buffer. To avoid any such incident, aspirate buffer from the diametrically opposite side of the tissue and add buffer on the wall of the microcentrifuge tube, away from the tissue.

**Pause point:** The E14.5 FLs, in fresh 1× PBS, can be stored at 4°C (for up to 2 months) before proceeding with further steps of the protocol.

16. Before cryo-block preparation, cryo-protect the FL tissue using 30% sucrose solution. Remove 1× PBS, add 1.5 mL 30% sucrose and place the 2 mL microcentrifuge tube on the tube revolver at 24°C–26°C for 30 min.

***Note:*** Sucrose will replace a part of the water molecules and prevent crystal formation during snap-freezing in the subsequent step.

17. After 30 min, transfer the tubes to 4°C, on a stand, for overnight (∼16 h) cryo-protection.

**CRITICAL:** At the start of cryo-protection, the FL floats on the surface of 30% sucrose solution. FL sinking to the bottom of the tube marks the end point of cryo-protection.

***Note:*** Certain antibody paratopes are considerably more predisposed to a particular conformation of epitopes. In such cases, the native epitopes available within the tissues cannot be efficiently identified by the antibodies. However, treatment with alcohols (like MeOH or EtOH) might bring about sufficient changes to the native epitopes, enabling optimum recognition. For example, while performing immunohistochemistry with Lyve-1 AF488 (Table 1), FLs would have to be fixed with 4% PFA for 30 min on ice, followed by three 30 min 1× PBS washes. At the end of the washes, the livers would have to be dehydrated with MeOH in a graded manner; 50% MeOH for 10 min and then 100% MeOH for another 10 min, followed by sucrose gradient and further steps leading to immunohistochemistry. Lyve-1 AF488 staining will not be optimal without the MeOH dehydration steps. This makes combining Lyve-1 AF488 with other antibody stainings relatively more challenging.

***Note:*** Usually, MeOH dehydration steps are avoided to prevent change of epitope conformation for other sensitive antigens (especially when performing HSC staining) within the tissue.

**Figure 3:**
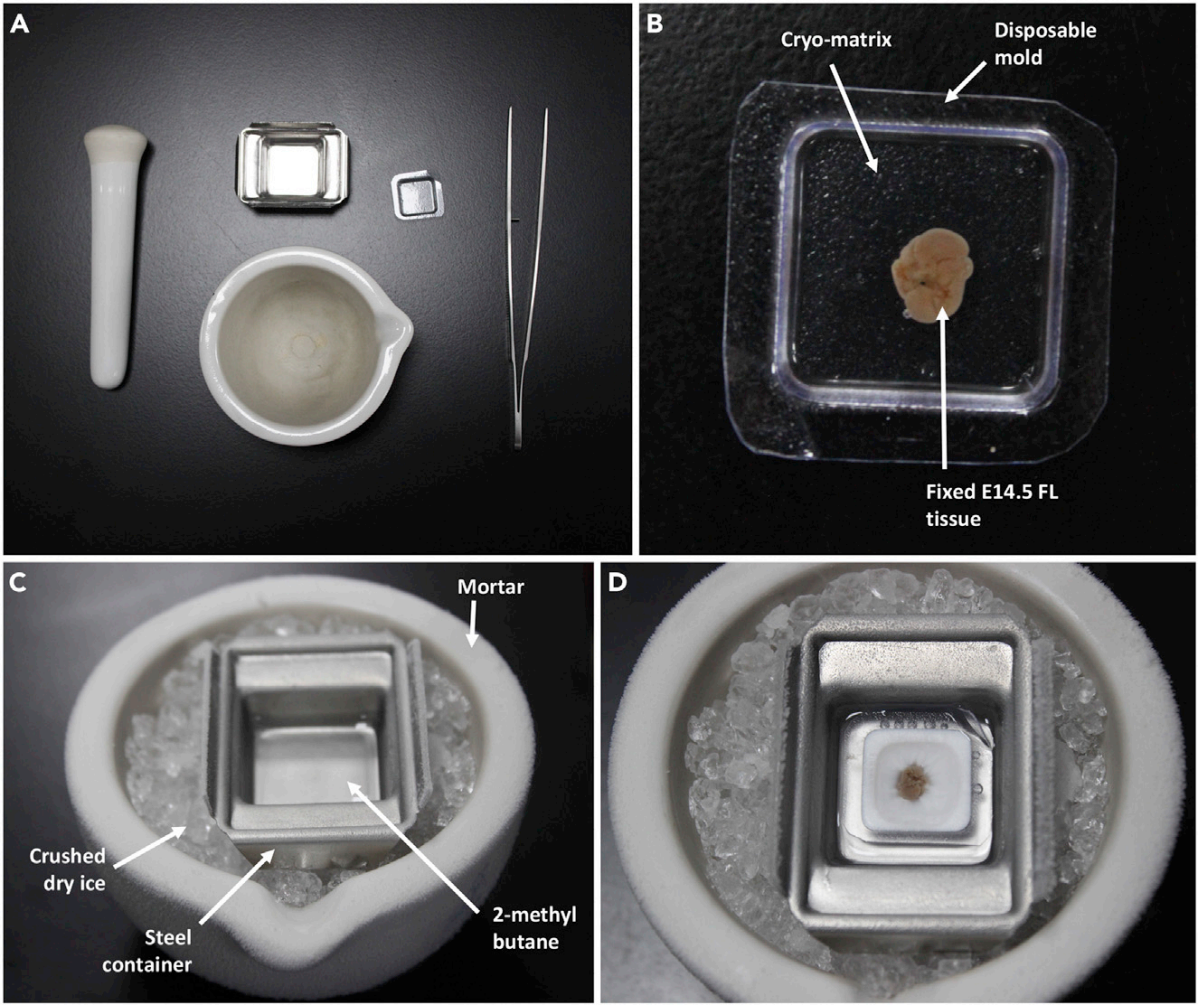
Preparation of E14.5 fetal liver cryo-block (A) Paraphernalia used for making cryo-block; clockwise from top: steel mold/ container, disposable plastic mold, a pair of Graefe forceps, mortar, pestle. (B) Fixed E14.5 FL sample immersed in PolyFreeze^TM^ cryo-matrix within disposable cryo-mold. (C) Set-up for indirect flash-freezing of FL tissue. (D) Flash-freezing E14.5 FL sample.

18. Put together the set-up for preparation of the cryo-mold (Figure 3A). Take a disposable mold (15 × 15 × 5 mm), fill it up with approximately 1.5 mL PolyFreeze^TM^ cryo-matrix and set it aside. Take a mortar and pestle and crush some dry ice. Place a stainless steel mold (24 × 24 × 10 mm), having approximately 2 mL inert 2-methyl butane, on the dry ice (Figure 3, Methods Video S3).

**Table 1:** Optimized working concentration and incubation time for antibodies used to label E14.5 FL cryo-sections

Sl. No.	Antibody	Clone	Working conc.	Incubation time
1.	CD31	–	1.5:300	16–20 h at 4°C
2.	Sca-1 FITC	D7	3.5:300	20–24 h at 4°C
3.	Ter119 AF647	TER-119	0.5:300	2 h at 24°C–26°C
4.	B220 APC	RA3-6B2	2.5:300	2 h at 24°C–26°C
5.	CD3e AF647	17A2	4:300	2 h at 24°C–26°C
6.	Gr-1 APC	RB6-8C5	2:300	2 h at 24°C–26°C
7.	F4/80 biotin	BM8	1.5:300	2 h at 24°C–26°C
8.	CD41 biotin	MWReg30	0.4:300	2 h at 24°C–26°C
9.	CD48 APC	HM48-1	2:300	2 h at 24°C–26°C
10.	Lyve-1 AF488	ALY7	2:300	16–20 h at 4°C
11.	Sca-1 biotin	177228	4:300	20–24 h at 4°C
12.	Ter119 PE	TER-119	1.25:300	2 h at 24°C–26°C
13.	B220 PE	RA3-6B2	4:300	2 h at 24°C–26°C
14.	CD3e PE	145-2C11	4:300	2 h at 24°C–26°C
15.	Gr-1 PE	RB6-8C5	2:300	2 h at 24°C–26°C
16.	F4/80 APC	BM8	4:300	16–20 h at 4°C
17.	CD41 PE	MWReg30	1:300	2 h at 24°C–26°C
18.	CD48 PE	HM48-1	4:300	2 h at 24°C–26°C
19.	Alexa Fluor® 647 Streptavidin	–	0.5:300	1 h at 24°C–26°C
20.	Alexa Fluor® 647 AffiniPure Goat Anti-Armenian Hamster IgG (H+L)	–	0.5:300	1 h at 24°C–26°C
21.	Alexa Fluor® 594 AffiniPure Fab Fragment Donkey Anti-Goat IgG (H+L)	–	0.5:300	1 h at 24°C–26°C
22.	Alexa Fluor® 647 AffiniPure Fab Fragment Donkey Anti-Goat IgG (H+L)	–	0.5:300	1 h at 24°C–26°C

**CRITICAL:** Avoid introducing bubbles by maintaining a continuous flow of the cryo-matrix while filling up the disposable mold. Bubbles that are near the specimen might cause the sections to tear during cryo-sectioning. Bubbles can be dispensed with the help of a pipette-tip.

***Note:*** The steel mold is placed on the dry ice to facilitate better conduction and bring down the temperature of the inert medium quickly.

***Note:*** Indirect snap-freezing method is used, keeping an inert medium between the dry ice and the sample, to avoid exposing the tissue to very harsh temperatures and achieve a more controlled rate of freezing (Figure 3C).

19. While the 2-methyl butane cools down (30–45 s), gently place the cryo-protected FL in PolyFreeze^TM^, immersing it completely within the cryo-matrix (Figure 3B).
20. Once the 2-methylbutane is cold, use a pair of forceps to place the mold, containing the FL, floating on top of the cold 2-methyl butane (Caution: do not submerge it). As it cools down, the cryo-matrix will start solidifying, changing from a transparent jelly to a solid white form (Figure 3D).
21. After the entire cryo-matrix has solidified, transfer the molds containing the cryo-blocks to −80°C for storage (Methods Video S3).

**Pause point:** The cryo-blocks can be stored at −80°C until the next steps in the protocol are carried out.

Methods Video S3. Preparation of cryo-block and storage, related to steps 18, 19 and 20, 21

### Cryo-sectioning and immunohistochemistry

**Timing: 2–4 days**

This section contains a step by step detailed procedure for obtaining 50 μm-thick cryo-sections of fixed E14.5 FLs and their staining by immunohistochemistry.

22. Keep the mold containing the cryo-block inside the cryotome for at least 30 min before taking cryo-sections. This will help raise the temperature from −80°C to −28°C.

**CRITICAL:** Cryo-sections are taken at −28°C. Obtaining cryo-sections immediately after taking the cryo-blocks out of −80°C might lead to brittle broken sections.

***Note:*** The cryotome temperature has to be maintained at −28°C. The paraphernalia required for taking cryo-sections like fine brush, cryotome blade, pair of Graefe forceps, should be maintained at −28°C as well, inside the cryotome.

23. Take a 24-well cell-culture plate and wrap the lid and the bottom half, separately, with aluminum foil. This plate will be used for performing immunohistochemistry and the aluminum foil would prevent light from entering the plate.

**CRITICAL:** The primary and secondary antibodies used in this protocol are fluorophore tagged. Care should be taken to allow minimum direct light exposure to preserve signals, as much as possible.

24. Label the wells where the immunohistochemistry experiments will be carried out. Fill these wells with 1.5 mL 1× PBS.

***Note:*** The described method is based on immuno-staining of free-floating sections of the FL tissues. While it can be applied to sections taken directly on glass slides, immunohistochemistry on free-floating sections results in more uniform staining, perhaps due to an enhanced antibody penetration. Hence, thicker sections can be labeled and imaged for better 3D characterization of the tissue. In addition, free-floating sections also allowed more effective washing, reducing background signals.

25. Once the temperature of the cryo-block has balanced with the cryotome (∼30 min), detach the cryo-block from the mold by pressing gently underneath the mold (Methods Video S4).

**CRITICAL:** Do not handle the cryo-block with hand, directly. Heat from the hand will melt the cryo-block. Use cold Graefe forceps to hold the cryo-block.

26. Pour approximately 1 mL PolyFreeze^TM^ onto a chuck, kept at 24°C–26°C. Put the chuck inside the cryotome and quickly place the cryo-block on the PolyFreeze blob, sticking it to the chuck before the cryo-matrix solidifies (Methods Video S4).

***Note:*** Press the cryo-block down gently, on the chuck, using horizontally placed Graefe forceps. This is done to obtain a properly aligned cryo-block-chuck assembly.

**Figure 4:**
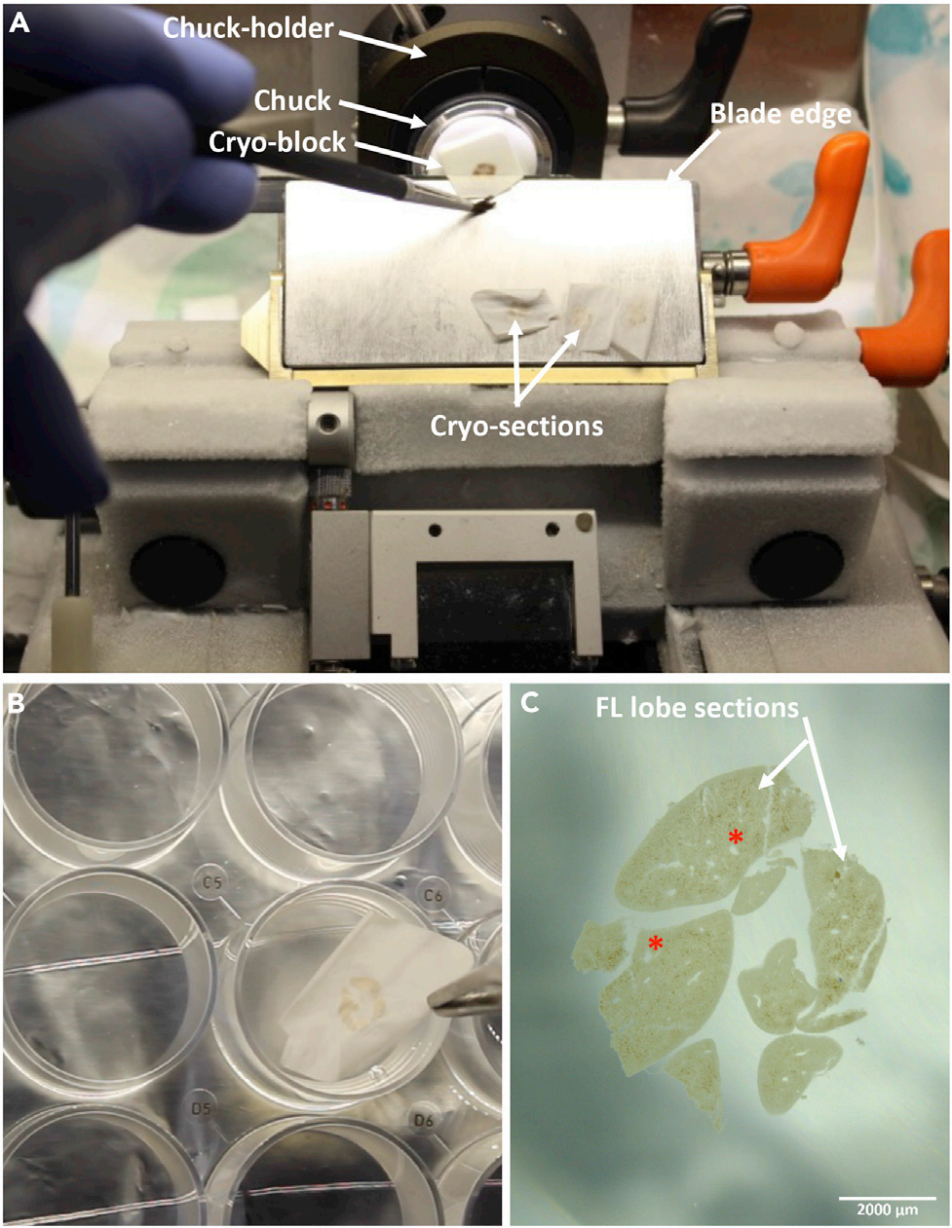
Cryosectioning E14.5 fetal liver tissue (A) Set-up inside the cryotome. (B) Transferring FL cryo-section into a 24 well cell culture plate. (C) 50 μm FL section, as observed under a stereo zoom microscope; asterisk (∗) denotes ovoid/ circular shaped blood vessels. Scale bar: 2000 μm.
28. Set the trimming thickness on the cryotome at 50 μm. Place the cryotome blade in the blade-holding slot and tighten the screws. Rotate the cryotome wheel handle in a clock-wise manner to start obtaining sections.

***Note:*** The movement of the cryotome wheel should be unidirectional (i.e., clockwise only), steady and continuous. This would ensure better quality of sections.

***Note:*** Initial trimming is done to get rid of the excess layers of cryo-matrix before reaching the sample.

27. After the cryo-matrix between the cryo-block and the chuck has solidified completely (∼2 min), insert the chuck in the chuck holder and tighten the screws to keep the sample in position (Figure 4A).

**CRITICAL:** Use separate blades for trimming and sectioning purposes. An older blade can be used for trimming and newer one for sectioning. This ensures that the blade used for sectioning is free of dents, which would result in better sections.

29. Switch to sectioning mode when the sample starts becoming visible through thinner layers of cryo-matrix. Set the sectioning thickness at 50 μm. Change the trimming blade to sectioning blade.
30. Rotate the cryotome wheel handle slowly and steadily while using a fine brush to pull out single cryo-sections from the edge of the cryotome blade (Methods Video S4).

**CRITICAL:** While pulling the cryo-sections, avoid putting the brush bristle on the tissue area of the section as it would damage the sample.

31. Bring the aluminum foil covered 24-well cell culture plate within the hood of the cryotome and quickly transfer the cryo-section into one of the wells, filled with 1× PBS, using a pair of Graefe forceps. This is done by carefully touching the section on the surface of 1× PBS. The cryo-matrix will immediately dissolve into the 1× PBS and the section will remain afloat (Figure 4B, Methods Video S4).

***Note:*** Sections that do not remain afloat, or are partially submerged in buffer, are nicked. For preventing nicked sections, check troubleshooting 1.

**CRITICAL:** Keep the section away from the wall of the well as the section would get stuck on the wall, leading to loss of tissue.

32. Discard the 1× PBS, containing dissolved PolyFreeze^TM^, and add 500 μL 1× PBST. This wash removes the cryo-matrix and starts permeabilizing the thick tissue.

***Note:*** Repeat steps 30–32 to obtain cryo-sections for all the immunolabeling experiments to be performed.

***Note:*** At a plane of the block where the E14.5 FL has more than one lobe, the section might split into the individual lobes after being floated in 1× PBS. This will not hamper immunolabeling. However, extra care should be taken to maintain the sections (from the different lobes) in buffer.

**CRITICAL:** Use 200 μL tip for changing buffers for better control of the floating tissue. Add buffers on the wall of the well to prevent agitation of the floating section.

**CRITICAL:** The tissue, at no point, should be left dry.

33. After obtaining the requisite number of sections, observe the sections under a stereo zoom microscope.

***Note:*** The FL vasculature has been defined to be an important niche component for HSCs. The first few sections obtained, while cutting through a new block of E14.5 FL, are usually devoid of prominent vasculature. Prominent vasculatures appear as circular or ovoid shaped gaps within the FL tissue, when observed under a stereo zoom microscope. Presence of such structures can be confirmed during this preliminary observation (Figure 4C).

***Note:*** The presence of nicked or partially brittle sections can also be detected under the stereo zoom microscope. Such sections should not be used for immunolabeling.

34. Use a thin film of cryo-matrix to cover the exposed tissue in the cryo-block. This prevents tissue dehydration from the exposed end. After the PolyFreeze^TM^ solidifies, store the remaining block on the chuck, within a labeled zip-lock bag, inside −80°C for future use.

***Note:*** Each time a used cryo-block is trimmed and sectioned, a part of the initial tissue is lost to obtain a leveled complete plane of the sample. To minimize this loss, the orientation of sectioning can be marked so that the angle of the blade and the angle of the used block can be aligned before re-starting the sectioning process. Also, performing experiments in batches can further help reduce this loss.

35. Transfer the 24-well cell culture plate, having the floating FL sections, on ice. Place this on the 2D rocker at 15 rpm for 5 min.
  ***Note:*** In all the succeeding steps, other than buffer changes and additions, the sections would have to be placed on the 2D rocker to facilitate proper mixing.
  ***Note:*** Sections can remain in 1× PBST until all the sections have been procured. However, the minimum duration of 1× PBST wash is 5 min (step 32).
  ***Optional:*** At the end of 5 min, discard 1× PBST and perform biotin blocking for the sections that would require Streptavidin conjugated fluorophore (secondary antibody) addition in subsequent steps of the experiment.
  a. Add 300 μL avidin to the sections and incubate at 24°C–26°C for 15 min.
  b. After 15 min, remove avidin and wash the sections with 300 μL 1× PBST for 5 min at 24°C–26°C.
  c. Discard the wash buffer and add 300 μL biotin and incubate at 24°C–26°C for 15 min.
  d. After 15 min, discard biotin and wash the sections for 5 min with 300 μL 1× PBST, at 24°C–26°C.
36. Prepare the optimal working concentration of primary antibody in 1× PBST. The working concentrations used for different primary antibodies have been listed in Table 1. Remove the wash buffer and add primary antibody to the sections and incubate for 2 h at 24°C–26°C or 16–24 h at 4°C, according to the conditions optimized for individual antibodies (refer to Table 1) (Methods Video S4).

***Note:*** The final volume of the working concentration for primary antibodies is 300 μL.

**CRITICAL:** This immunolabeling protocol involves the usage of several primary and secondary antibodies. These antibodies were selected after screening a number of commercially available products against each marker. Additionally, their working concentration as well as incubation period was optimized carefully to avoid non-specific signals (Table 1). These products were tested thoroughly for the objective described. Table 1 also lists alternative reagents that have been tested, and successfully used, for optimal outcome of this method. However, changing the target cell type or incorporation of additional/alternative markers may require additional optimization of the antibodies as well as working concentration and time of incubation. Optimization would have to be performed for any new antibody used to label a new cell type of choice.

***Note:*** For CD31, Lyve-1 AF488 and F4/80 APC, signals are obtained 2 h post-incubation at 24°C–26°C, however, optimum results are obtained by 16–20 h incubation at 4°C. For Sca-1 FITC and Sca-1 biotin, 20–24 h incubation at 4°C is integral for securing signals.

37. After primary antibody incubation, wash the sections with 1× PBST twice, for 5 min each, at 24°C–26°C.
38. Prepare the working concentration for the secondary antibodies in 1× PBST. The working concentrations used for different secondary antibodies have been listed in Table 1. Remove the wash buffer and add secondary antibody to the sections and incubate for 1–2.5 h at 24°C–26°C, according to the conditions optimized for individual antibodies (refer Table 1).

***Note:*** The final volume of the working concentration for secondary antibodies is 300 μL.

39. After secondary antibody incubation, wash the sections with 1× PBST for 5 min at 24°C–26°C.

***Note:*** Repeat steps 36–39 for completing antibody additions for all pairs of primary and secondary antibodies of the experimental combination.

**Figure 5:**
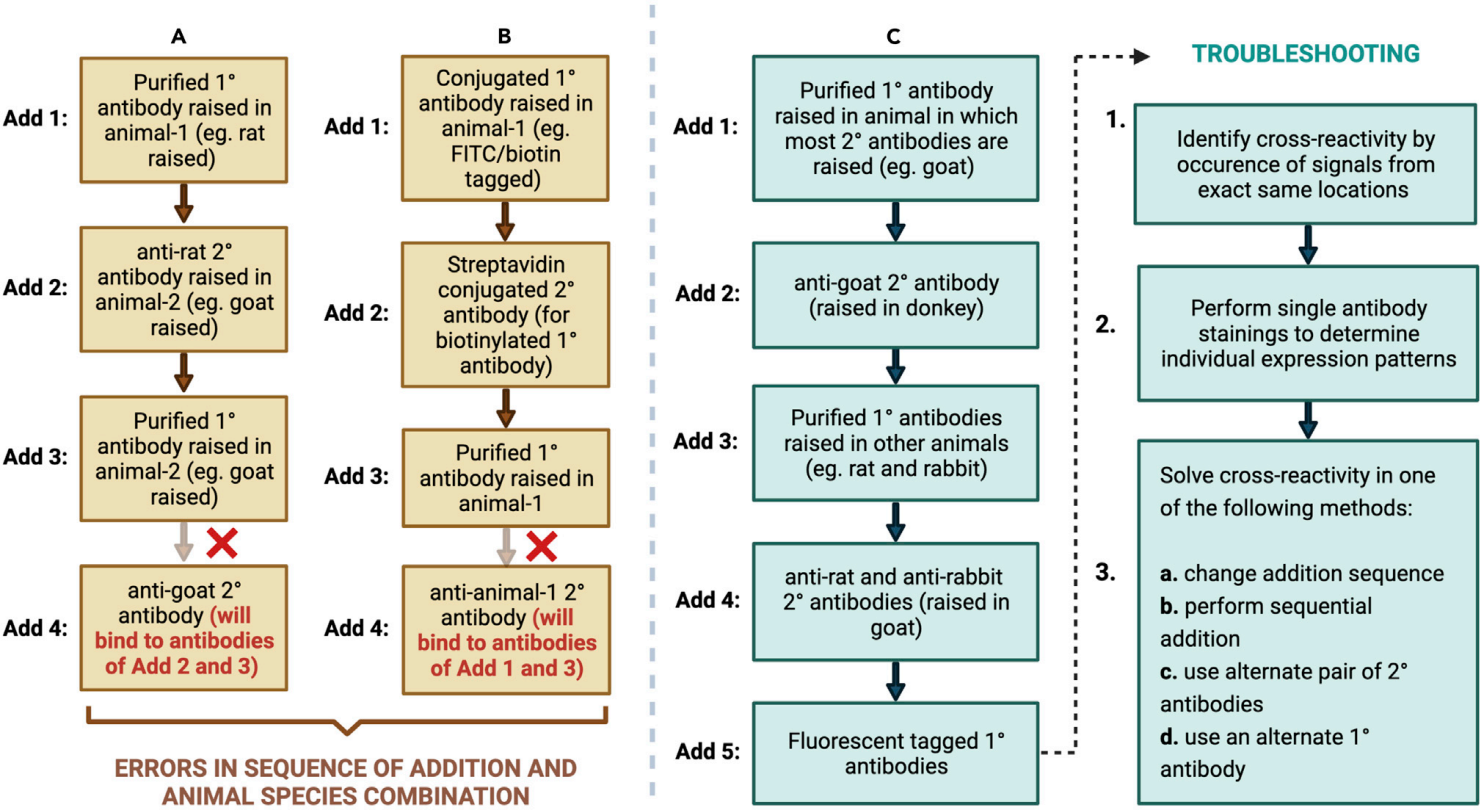
Staining panel to avoid antibody cross-reactivity (A and B) Flowcharts showing possible errors made in staining panels. (C) Flowchart elaborating ideal staining panel with troubleshooting techniques.

**CRITICAL:** Depending on the experimental combination and animal sources, primary antibodies could be added together or sequentially. Sequential addition of antibodies is performed in order to avoid cross-reactivity of antibodies or binding of secondary antibodies to multiple primary antibodies (Figure 5).

Methods Video S4. Immunolabeling and microscopy, related to steps 25, 26, 27, 28, 30, 31, 36, 42, 43, 45, 46, 48, 49 and 56

40. Discard wash buffer and add 300 μL working concentration of Hoechst 33342, for 5–10 min at 24°C–26°C, for counterstaining.

**CRITICAL:** Perform Hoechst counterstaining only if secondary antibody in the excitation and emission range of Hoechst dye (AF405) is absent from the experiment combination.

***Optional:*** This protocol can alternatively be followed for performing E14.5 FL HSC staining in combination with up to two niche components, provided the niche components are identified by single markers.

The sequential addition steps for staining HSCs in combination with pan-endothelium (marked by CD31) have been listed below in Table 2.

***Note:*** In the experiment combination above (Table 2), anti-Armenian Hamster 2° antibody (Add 4) is used to enhance the signal intensity of CD48 APC 1° conjugated antibody (Add 3).

***Note:*** Biotin blocking and requisite washes are done for the aforementioned experimental combination.

***Note:*** These steps can be extrapolated and developed further to perform more combinations of similar experiments.

**CRITICAL:** For the experiment mentioned above, anti-goat AF594 2° antibody (Add 2) is raised in donkey and anti-Armenian hamster AF647 2° antibody (Add 4) is raised in goat. To prevent anti-goat AF594 from binding to goat raised anti-Armenian hamster AF647, the former is added first in the sequential steps **(**Figures 5A and 5C**)**.

**Table 2:** Sequence of addition for performing HSC CD31 combination experiment in E14.5 FL tissue section

Addition step	Antibodies/ dye added	Incubation time & conditions
Add 1 (1°)	CD31 purified (goat raised)	16–20 h at 4°C
Add 2 (2°)	anti-goat AF594 (donkey raised)	1 h at 24°C–26°C
Add 3 (1°)	CD48 APC (Armenian hamster raised) + CD41 biotin + F4/80 biotin	2 h at 24°C–26°C
Add 4 (2°)	anti-Armenian hamster AF647 (goat raised) + Streptavidin AF647	1 h at 24°C–26°C
Add 5 (1°)	Sca-1 FITC	20–24 h at 4°C
Add 6 (1°)	Ter119 AF647 + B220 APC + CD3e APC + Gr-1 APC	2 h at 24°C–26°C
Add 7	Hoechst	5–10 min at 24°C–26°C

Care should be taken to design experimental combinations and additions such that interferences and cross-reactivities stemming from animal sources can be avoided (Figures 5A and 5B). Figure 5 presents the sequential steps that can be taken to select correct antibodies for multiple markers in a staining protocol, and avoiding cross-reactivity.

### Mounting and storage

**Timing: 17 h**

In this section, the procedure for mounting and storing 50 μm thick E14.5 FL cryo-sections, post immunohistochemistry, has been described.

**Figure 6:**
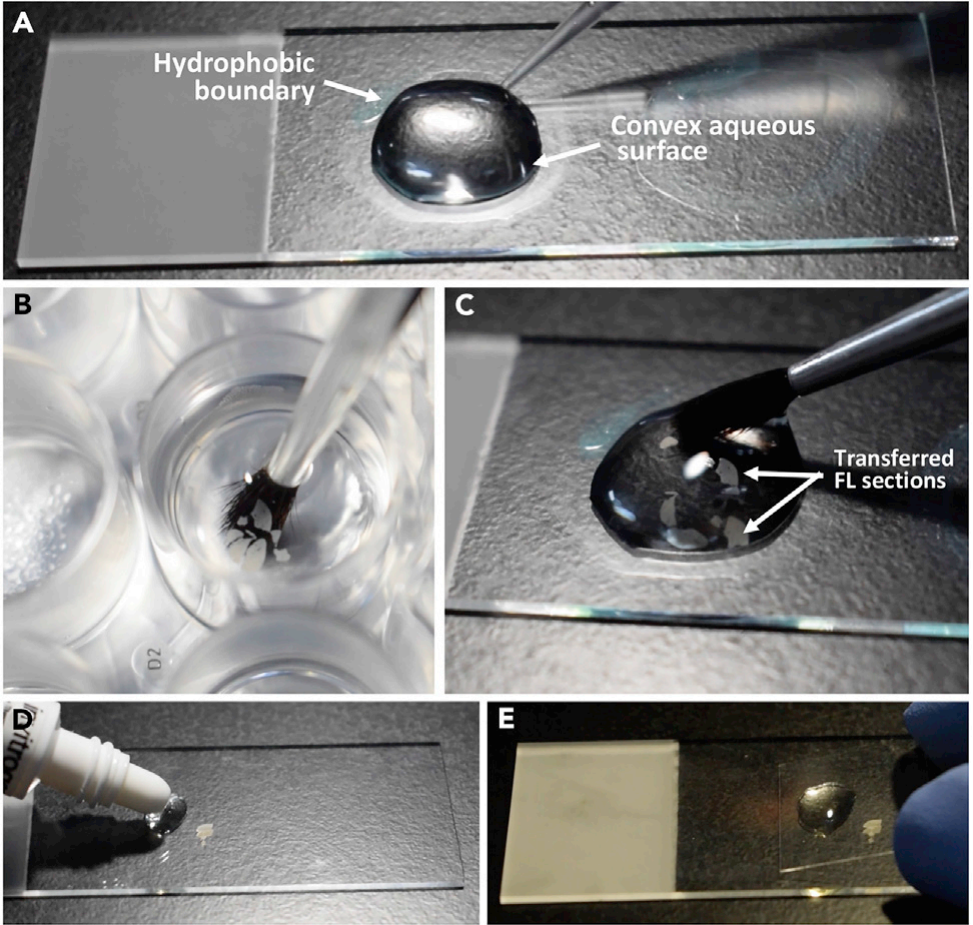
Mounting immunostained E14.5 fetal liver section (A) Frosted slide showing hydrophobic boundaries and a convex aqueous surface. (B) Lifting FL section with a fine brush. (C) Transferring FL section onto the convex aqueous surface. (D) Addition of mounting media slightly ahead of the FL section. (E) Lowering cover glass on the FL section.

***Note:*** The inner diameter of the hydrophobic boundary should approximately be 1.5 cm.

41. Take a frosted slide and wipe it with lint-free tissue paper. Lay it down on a black surface. The black surface will provide better contrast and help to clearly visualize the tissue section.
42. Use a PAP pen to draw circular hydrophobic boundaries on the frosted slide. Two to three circles can be drawn on one slide. Take care to position the circles away from the edge of the slide (Figure 6A, Methods Video S4).

43. After the boundaries are completely dry, add 100–200 μL 1× PBS inside the boundary to create a convex aqueous surface where the sections can be floated (Figure 6A, Methods Video S4).

**CRITICAL:** Adding 1× PBS while the boundary is wet would make the PAP pen ink form a film over the aqueous media. This would lead to non-specific fluorescence from the samples, during imaging.

44. Discard Hoechst (step 40) and add 2 mL 1× PBS into the sample well using a 200 μL tip. This would raise the tissue section from the bottom of the well.
45. Dip a wet, fine brush very carefully into the well and underneath the floating section without touching it. Once the bristles of the brush are completely immersed in 1× PBS and the section is stable on the surface, gently lift the brush out of the well such that the section layers the outer surface of the brush (Figure 6B, Methods Video S4).

**CRITICAL:** Make sure that the brush is smooth, fine and uniform. Brushes with individual bristles sticking out would make tears in the section.

46. Hold the brush horizontally and gently roll the section off onto the aqueous convex surface built on the slides. Carefully remove the 1× PBS from within the hydrophobic boundary using a 200 μL tip (Figure 6C, Methods Video S4).
47. Use the pointed end of a folded lint-free tissue paper to remove the hydrophobic boundary surrounding the section. Absorb the left-over 1× PBS as well, taking care not to wipe the tissue off.
48. After the tissue loses most of the moisture content, add a drop of Prolong Gold anti-fade mountant slightly ahead of the section. We avoid adding mounting media directly on the section because the sample might come off the slide when the nozzle of the mountant tube is raised from the surface of the slide. This is because the sections are placed on the slides after immunolabeling and are not effectively bound to the slides due to difference of charges (Figure 6D, Methods Video S4).
49. Place a coverslip on the drop of mounting media and let it spread across the edge before lowering it gently over the sample (Figure 6E, Methods Video S4).

***Note:*** After lowering the coverslip over the tissue, make sure that the mounting media spreads uniformly over the entirety of the section.

**CRITICAL:** Prevent the formation of bubbles. Bubbles that have been introduced can be removed with the help of 200 μL tip.

**CRITICAL:** Do not over-dry the sections before mounting them.

50. Carefully place the mounted slides in a dark area and leave them to dry at 24°C–26°C for approximately 16 h. After overnight drying, transfer the slides to 4°C until the samples are being imaged.

**Pause point:** Dried slides can be maintained in 4°C for 2–3 weeks before they are imaged. After this, the signals from some of the fluorophores will start diminishing significantly.

### Imaging

**Timing: 3–6 h per section**

This section mainly describes the settings that were used for imaging immunostained E14.5 FL sections, using Zeiss LSM 880.

51. Put a drop of Immersol^TM^ 518F over the coverslip, covering the area right above the tissue section. Plan-Apochromat 63×/1.40 Oil DIC M27 lens will be used for imaging the FL tissue sections.
52. Mount the slide on the slide holder of the microscope stage. Use the UV range light from the fluorescent lamp to coarsely focus the tissue section under 10× objective magnification.

***Note:*** Hoechst is excitable in the UV range and we utilize this nucleus staining dye to bring our section into focus. Alternatively, in the absence of Hoechst, any photo-stable fluorophore can be used to focus the sample.

53. After adjusting coarse focus, change the objective to Plan-Apochromat 63×/1.40 Oil DIC M27 lens and tune the fine focus knob until the nucleus appears clear. Move the stage in x-y plane to choose a region of interest, in this case an area having proper vasculature.
54. Scan the area of interest by going through all the channels and set the optimal signal strength by adjusting laser intensity and gain master. Denote the ‘start’ and ‘end’ z-steps of the total FL volume to be imaged (Methods Video S4).
55. Use the following settings for imaging:

Image size: 1024 × 1024 pixels.

Line average: 1.

Scan direction: Unidirectional.

Detector type: PMT.

Pinhole: 1 Airy Unit.

z-increment (step-size): 1 μm.

Scan speed: 6 Hz.

56. Set the emission capture range according to the following slider values and start imaging:

Channels: Ex_633 nm_: 636 nm–758 nm.

 Ex_561 nm_: 561 nm–635 nm.

 Ex_488 nm_: 492 nm–560 nm.

 Ex_405 nm_: 408 nm–487 nm.

***Note:*** Capture images from 10–15 different areas on the tissue section to ensure adequate sampling.

***Note:*** Alternatively, tile scanning can be done to take into account the entire area of the section and obtain complete perspective of the tissue slice. Tile scanning can be taken in combination with z-stack to acquire the entire volume of the FL tissue.

57. After completion of imaging, name the image files appropriately and save them in the designated folder.

**CRITICAL:** Avoid imaging the edges of the tissue as there will be non-specific binding of antibodies, leading to non-specific fluorescence from the area.

## Expected outcomes

**Figure 7:**
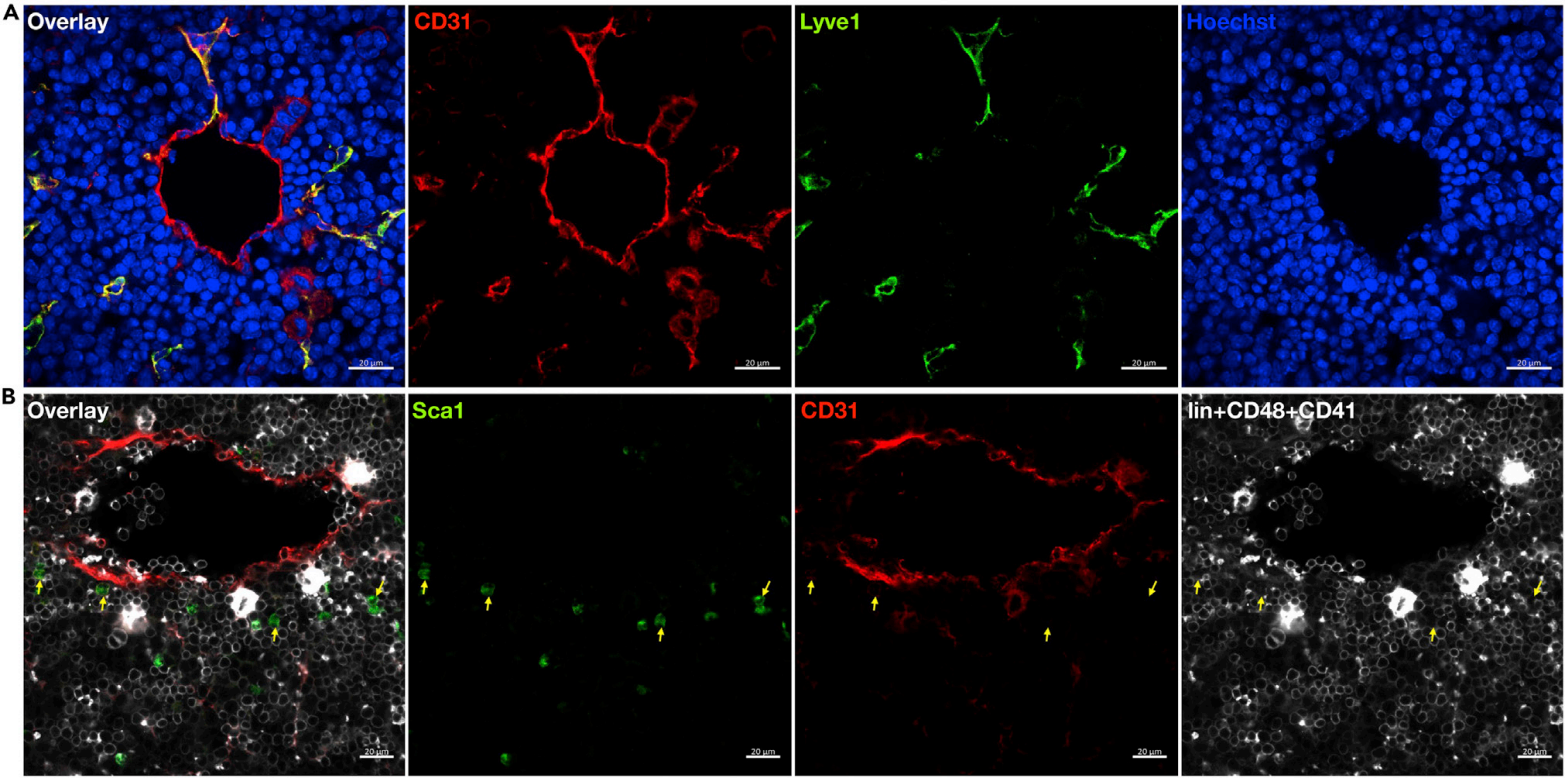
Z-projection through a stack of confocal micrographs of E14.5 fetal liver section (A) Representative image shows CD31^+^ pan-endothelium (red), Lyve-1^+^ sinusoidal endothelium (green) and Hoechst counter-stained nuclei (blue). (B) Representative image shows CD31^+^ pan-endothelium (red), 3^+^ cells (white) and Sca-1^+^ cells (green); Yellow arrows mark 3^-^ Sca-1^+^ HSCs. Scale bar: 20 μm.

This protocol can be used for immunolabeling and marking distinct cell types and structures present in the E14.5 murine FL. Further, in-depth three dimensional image analysis would help establish the spatial associations between various individual components. Using this protocol, we immunostained FL vasculature with pan-endothelial marker, CD31 (Garcia-Porrero et al., 1998; Albelda et al., 1991), and with sinusoidal endothelial marker, Lyve-1 (Nonaka et al., 2007) (Figure 7A), to understand the relative sinusoidal abundance in the tissue. Additionally, we used lineage antibody cocktail (Ter119^-^ Gr-1^-^ CD3e^-^ B220^-^ F4/80^-^) CD41^-^ CD48^-^ and Sca-1^+^ markers to identify HSCs within the FL tissue, in conjunction with CD31^+^ pan-endothelium (Figure 7B). Insight of the cells that build up the microenvironment and support FL HSCs can be investigated.

## Quantification and statistical analysis

This set of procedures allows the creation of pseudo-surfaces from structures or cells of interest and subsequently perform three dimensional distance analysis between the rendered surfaces. Images have been analyzed using Imaris, with the help of a MATLAB based Xtension that can be downloaded from an open source online repository (link redirecting to the repository can be found in key resources table).

### Image processing using Imaris

**Timing: variable (estimated to be 12–16 h per frame of interest)**

**Figure 8:**
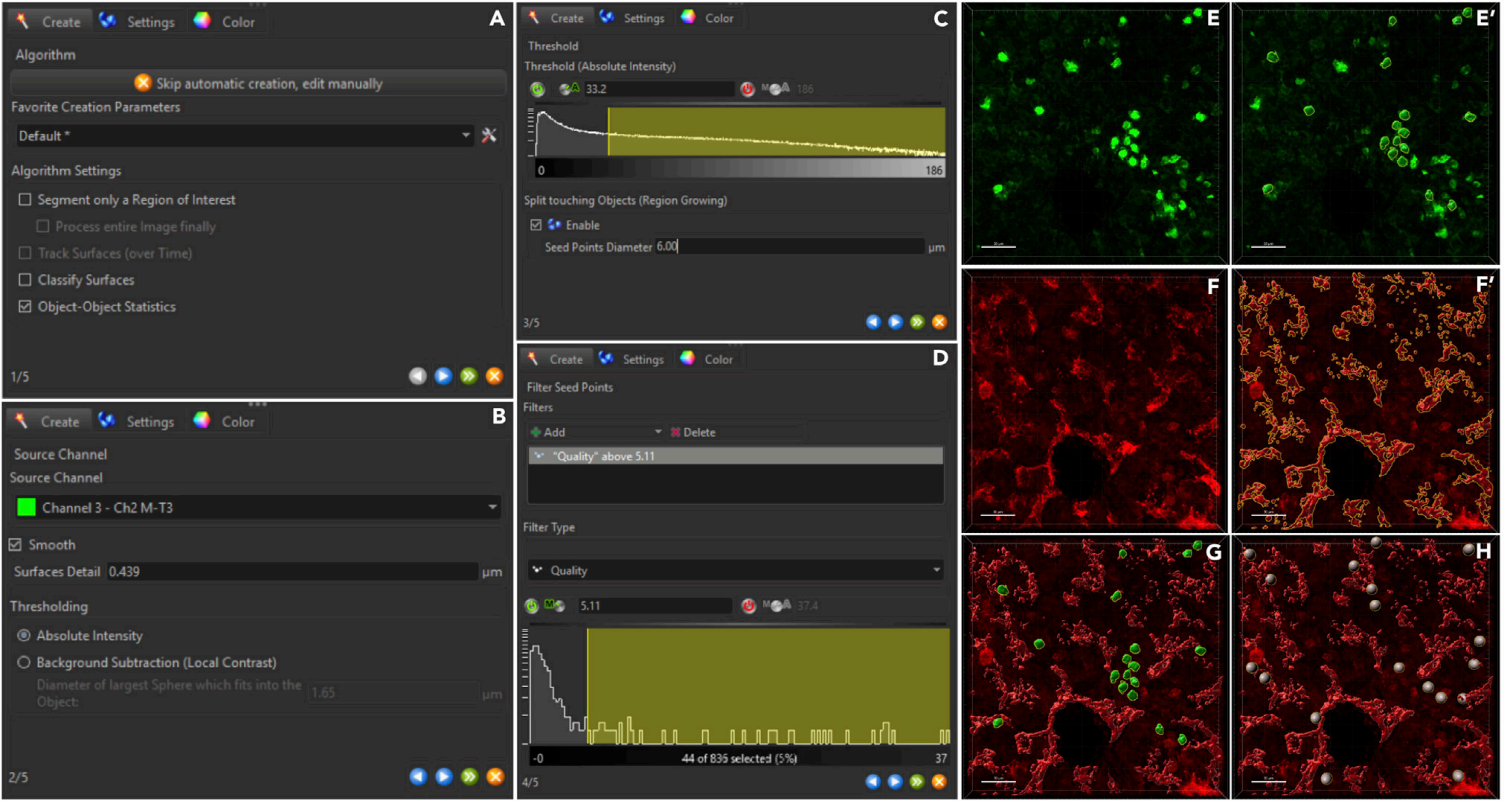
Workflow for pseudo-surface generation (A–D) Steps showing generation of pseudo-surfaces for HSCs. (E) Three dimensional volumetric rendition of Sca-1^+^ cells. (E′) Pseudo-surfaces generated against Sca-1^+^ cells; surfaces that were 3^-^(lin^-^CD41^-^CD48^-^) Sca-1^+^ were manually curated. (F) Three dimensional volumetric rendition of CD31^+^ vessels (pan-endothelium). (F′) Pseudo-surfaces generated against CD31^+^ vessels. (G) Shortest distances between the pseudo-surfaces of 3^-^Sca-1^+^ HSCs and CD31^+^ vessels were exported. (H) Shortest distances between several iterations of randomly generated dots and pseudo-surface of CD31^+^ vessels were exported. Scale bar: 30 μm.

In this segment, the “Surfaces” function of Imaris image analysis software will be used to generate pseudo-surfaces of vasculature and FL HSCs from 3D reconstructed volumes of the imaged tissue sections. The “HSC” surfaces (Sca-1^+^ only) identified will be manually curated to retain the ones that are negative for lineage, CD48 and CD41 markers (or 3^-^). Imaris in-built “shortest distance between surfaces” function will be used to obtain the three dimensional Euclidean distances between each HSC and the vessel lying closest to it. Random spheres, of similar size as the HSCs, will be generated as control, in multiple iterations, using a MATLAB XTension. The shortest distance between random spheres and vessel surfaces will be generated (Figure 8).

• Use Imaris File Converter to generate .ims files from .czi files (file format generated from Zen software).
• Open .ims file in Imaris: File → Open. Choose the required .ims file from the destination folder and select “Open”.
• Generating pseudo-surface for vasculature:
  ○ Deselect channels other than the one displaying vasculature under “Display Adjustment.”
  ○ Perform a surface object selection “Add new surfaces” under Surpass.
  ○ Double click to rename the added surface, e.g., CD31, for vasculature. Make sure that “Volume” is checked under Surpass.
  ○. Under Creation wizard, check “Object-Object Statistics” only and click next.
  ○ Choose the channel for vasculature under “Source Channel.” Check on “Smooth” and enter a “Surface Detail” value of 0.65 μm. Under “Thresholding” check “Background Subtraction (Local Contrast)” and enter a value of 1.85 μm under “Diameter of largest sphere which fits into the object.” Click next.
  ○ Under Threshold, move the slider to the left or manually decrease the value under “Threshold (Background Subtraction)” to increase the preview surfaces and move the slider to the right or increase the “Threshold (Background Subtraction)” value to decrease the preview surfaces. Click next.
  ○ In the next step, pseudo-surfaces can be fine-tuned further by adding filters like “Number of voxels above (a certain value)” or “Area.” Use the slider underneath or manually change the values to reach the desired pseudo-surface representation. Then press the finish button to terminate the creation wizard and obtain pseudo-surface against vasculature (Figures 8F and 8F′).
• Generating pseudo-surface for HSCs:
  ○ Deselect channels other than the one displaying “HSCs” under “Display Adjustment.” Deselect the vasculature pseudo-surface created as well.
  ○ Perform another surface object selection by clicking on “Add new surfaces” under Surpass.
  ○ Double click to rename the added surface, e.g., Sca-1, for HSCs.
  ○ Similarly, under Creation wizard, check “Object-Object Statistics” only and click on next (Figure 8A).
  ○ Choose the channel for HSC under “Source Channel.” Check on “Smooth” and do not change the default “Surface Detail” value. Under “Thresholding” check “Absolute Intensity” and click next (Figure 8B).
  ○ Under Threshold, move the slider left or right to set the representative preview pseudo-surfaces. Check “Enable” under “Split touching Objects (Region Growing)” and enter a “Seed Points Diameter” of 6 μm. Click next (Figure 8C).
  ○ In the next step, move the slider left or right under “Filter Seed Points” to incorporate one seed point at the center of each Sca-1^+^ cell. Click next (Figure 8D).
  ○ Previewed pseudo-surfaces can be fine-tuned further in this step. Use the slider under “Filter Surfaces” to remove any extra surface selected. Observe the splits that have been included in the clumped up Sca-1^+^ cells as a result of seed-points (water-shedding). Click on finish to terminate the creation wizard and obtain pseudo-surfaces generated against Sca-1^+^ cells (Figures 8E and 8E′).
• For either of the pseudo-surfaces generated, extra non-specific surfaces can be further removed by using the “Edit” tool. Select the pseudo-surface, then go to “Edit”, click on the extra surface that has to be deleted. Selection of the surface turns it from its pseudo-color to the default color, yellow. Click “Delete.”
• Manually toggle between “Slice” and “3D View” to hand-pick the Sca-1^+^ cells that are 3^-^. Delete the extra Sca-1^+^ 3^+^ pseudo-surfaces (Figure 8E′).
• Under the Sca-1 pseudo-surface generated, select “Statistics” tool and go to “Detailed.” Under “Detailed,” select “Specific Values.” In the drop-down box below, select “Shortest Distance to Surfaces Surfaces=CD31.” The shortest distances (Euclidean distance) between HSCs and vasculature will be obtained (Figure 8G).
• Export the Statistics generated by clicking on the Floppy like option at the bottom of the generated table. A .xls file will be created which can be saved at the required destination.
• Generation of Random Spheres:
  ○ Take the x, y and z dimensions (in μm) of the entire FL volume under consideration. Edit → Image Properties. Obtain the extent of x, y and z dimensions displayed under “Min” and “Max.” Generate equal number of randomized x, y, z co-ordinates, as the number of HSCs, within the limits of the x, y and z dimensions of the FL volume. Use the “RANDBETWEEN(Minimum value, Max value)” function in Microsoft Excel to generate random numbers between the known minimum and maximum values for each dimension (x, y and z). Enter the ranges for x, y and z in the first 3 columns respectively, and use “1” in the fourth column against time, which is constant. Drag down to obtain as many (x,y,z,t(1)) random co-ordinates as are necessary. It is important to note that these random co-ordinates remain random in a .xls format and on saving the files in .csv format, the property of random number generation is lost. However, these files would have to be converted into the .csv format in order to be identified by the MATLAB XTension. Make multiple such .csv files for the different iterations of random sphere generation. These are the input files for creation of random spheres.
  ○ Download the MATLAB XTension “Creating spots from .csv” from the open source online repository (link provided in key resources table) and save it in the MATLAB folder within Program Files.
  ○ For integrating the MATLAB XTension in Imaris, go to File → Preference → Custom Tools. Specify the destination of the downloaded MATLAB XTension by adding it in the XTension folder field.
  ○ For running the MATLAB XTension, go to Image Processing → Spots Function → Create Spots from CSV → select the input .csv file → Enter spot radius “5.” Random spheres will be generated within the specified FL volume (Figure 8H).
    ***Note:*** Spheres generated within the vasculature cannot be considered and the simulations generating spheres within the voids of vessels would have to be disregarded.
    ***Note:*** Random sphere iterations are run for a minimum of 100 times per image frame.
  ○ Select “Spots from XT Create Spots from CSV” under Surpass Scene. Go to “Edit” tool and check “Object-Object Statistics.”
  ○ Select “Statistics” tool → “Detailed” → “Specific Values” → “Shortest Distance to Surfaces Surfaces=CD31.” This will generate a table of shortest distances (Euclidean distances between the random spheres and the vasculature).
  ○ Export the Statistics generated and save it within the required destination folder.
    ***Note:*** The distances obtained would then be classified under bin sizes of 5 μm, e.g., 0–5 μm, 5–10 μm, 10–15 μm and so on. Percentage of HSCs scored (y-axis) would be plotted against the shortest distance values (x-axis). Kolmogorov-Smirnov test will be used to compare the probability distributions generated from the distance data.

## Limitations

The immunolabeling technique described, although seemingly straightforward, has some limitations associated with it. The most critical one of them all is antibody cross-reactivity. Antibody combinations that can be paired with one another without giving rise to any cross-reactivity, need to be identified (Figure 5). The antibody list provided in Table 2 is an example of a curated cocktail that would specifically bind to their target proteins in the tissue without interfering with the other markers. Ways to troubleshoot issues with cross-reactivity has been discussed in the section below.

Furthermore, the markers (lin^-^CD41^-^CD48^-^Sca-1^+^) that were used to denote FL HSCs ensure a purity of approximately 75.02 ± 5.35% (data not shown). We use a confocal microscope with the ability to distinctly image only up to four different channels, without significant bleed through. Therefore, HSC denotation was restricted to two channels in order to accommodate the immunolabeling of putative niche cells. However, high end microscopes with more laser options and better spectral separation can be used to include more number of fluorophores, using the same protocol.

Moreover, the maximum depth of imaging achieved was limited to 20–25 μm, although the experiments were carried out on 50 μm thick sections. This is because the penetration capability of the different antibodies used are variable, the depth of imaging being confined by the antibody having the least penetrance.

## Troubleshooting

### Problem 1

The sections are nicked or torn along a line (step 30).

### Potential solution

While performing cryo-sectioning, the rotation of the wheel should be steady. Importantly, making halts during sectioning, while the tissue is directly exposed to the blade edge, would impart fine gashes on the section which would ultimately appear nicked once floated on 1× PBS.

### Problem 2

Cross-reactivity of antibodies; fluorescent signal appearing from the same sites for two unrelated primary or secondary antibodies (Figure 5).

### Potential solution

Cross-reactivity of antibodies can happen due to a lot of reasons. Firstly, antibody cocktails should be prepared such that cross-reactivity can be avoided due to erroneous combinations. Complex combinatorial experiments generally use antibody cocktails wherein primary and secondary antibodies are raised in several animal species. Care should be taken to formulate accurate sequential addition steps to avoid inadvertent invalid animal combinations.

From our experience with multiple antibody combinations, we have observed that certain pairs of secondary antibodies cannot be introduced in the cocktail together. Although theoretically these combinations work, it is ideal to demarcate these antibodies and not use them in the same experiment.

To troubleshoot an experiment where two antibodies seem to be appearing from the exact same locations, single antibody staining should be performed to delineate the precise stainings from the individual components of the experiment. Carrying out sequential addition of antibodies, one after the other, also helps resolve cross-reactivity issues.

### Problem 3

FL sections are brittle. Sections disintegrate after being transferred to 1× PBS (steps 13–15).

### Potential solution

FL sections are usually brittle when the tissues get over-fixed. Meticulous time-keeping for fixation is a pre-requisite of immunolabeling experiments.

### Problem 4

Weak fluorescent signals.

### Potential solution

Weaker fluorescent signals might be indicative of an array of underlying issues. Firstly, the permeabilization might not be optimum for the antibody in question. The percentage of Tween 20 used to prepare PBST, in this protocol, is 0.1%. For selected antibodies, this percentage can be increased to check whether better signals can be achieved.

Second, the incubation time for a particular antibody might not be sufficient. Additionally, increasing the antibody concentration might also help rectify this issue.

### Problem 5

Secondary antibody precipitates observed while imaging the FL sections.

### Potential solution

This is usually indicative of insufficient wash steps to remove excess secondary antibodies. To avoid this, it should be ensured that the sections are freely floating during the entire duration of the experiment, including the wash steps. Increasing the time of wash steps can also prove to be useful.

## Resource availability

### Lead contact

Further information and requests for resources and reagents should be directed to and will be fulfilled by the lead contact, Satish Khurana (satishkhurana@iisertvm.ac.in).

### Materials availability

This study did not generate new unique reagents.

## Data Availability

This study did not generate/analyze any datasets or code.
